# A Data Mining-based Prognostic Algorithm for NAFLD-related Hepatoma Patients: A Nationwide Study by the Japan Study Group of NAFLD

**DOI:** 10.1038/s41598-018-28650-0

**Published:** 2018-07-11

**Authors:** Takumi Kawaguchi, Katsutoshi Tokushige, Hideyuki Hyogo, Hiroshi Aikata, Tomoaki Nakajima, Masafumi Ono, Miwa Kawanaka, Koji Sawada, Kento Imajo, Koichi Honda, Hirokazu Takahashi, Kohjiroh Mori, Saiyu Tanaka, Yuya Seko, Yuichi Nozaki, Yoshihiro Kamada, Hideki Fujii, Atsushi Kawaguchi, Tetsuo Takehara, Mikio Yanase, Yoshio Sumida, Yuichiro Eguchi, Masataka Seike, Masato Yoneda, Yasuaki Suzuki, Toshiji Saibara, Yoshiyasu Karino, Kazuaki Chayama, Etsuko Hashimoto, Jacob George, Takuji Torimura

**Affiliations:** 1 Department of Medicine, Kurume University School of Medicine, Kurume, Japan; 2 Department of Internal Medicine and Gastroenterology, Tokyo Women’s Medical University, Tokyo, Japan; 3 Department of Gastroenterology and Hepatology, JA Hiroshima General Hospital, Hatsukaichi, Japan; 4 Department of Gastroenterology and Metabolism, Applied Life Science, Institute of Biomedical and Health Sciences, Hiroshima University, Hiroshima, Japan; 5 Department of Hepatology, Sapporo Kosei General Hospital, Sapporo, Japan; 6 Department of Gastroenterology and Hepatology, Kochi Medical School, Nankoku, Japan; 7 Department of General Internal Medicine2, General Medical Center, Kawasaki Medical School, Okayama, Japan; 8 Division of Gastroenterology and Hematology/Oncology, Department of Medicine, Asahikawa Medical University, Asahikawa, Japan; 9 Department of Gastroenterology, Nayoro City General Hospital, Nayoro, Japan; 10 Department of Gastroenterology and Hepatology, Yokohama City University School of Medicine, Yokohama, Japan; 11 Department of Gastroenterology, Faculty of Medicine, Oita University, Yufu, Japan; 12 Internal Medicine, Saga University faculty of Medicine, Saga, Japan; 13 Liver Center, Saga University Hospital, Saga, Japan; 14 Center for Digestive and Liver Diseases, Nara City Hospital, Nara, Japan; 15 Department of Gastroenterology and Hepatology, Kyoto Prefectural University of Medicine, Kyoto, Japan; 16 Division of Hepatology and Pancreatology, Department of Internal Medicine, Aichi Medical University, Nagakute, Japan; 17 Department of Gastroenterology, National Center for Global Health and Medicine, Tokyo, Japan; 18 Department of Gastroenterology and Hepatology, Osaka University, Graduate School of Medicine, Suita, Japan; 19 Department of Hepatology, Osaka City University, Graduate School of Medicine, Osaka, Japan; 20 Department of Gastroenterology and Hepatology, Osaka City Juso Hospital, Osaka, Japan; 21 Center for Comprehensive Community Medicine Faculty of Medicine, Saga University, Saga, Japan; 22 Storr Liver Centre, Westmead Institute for Medical Research, Westmead Hospital and University of Sydney, Sydney, NSW Australia

## Abstract

The prognosis of patients with nonalcoholic fatty liver disease-related hepatocellular carcinoma (NAFLD-HCC) is intricately associated with various factors. We aimed to investigate the prognostic algorithm of NAFLD-HCC patients using a data-mining analysis. A total of 247 NAFLD-HCC patients diagnosed from 2000 to 2014 were registered from 17 medical institutions in Japan. Of these, 136 patients remained alive (Alive group) and 111 patients had died at the censor time point (Deceased group). The random forest analysis demonstrated that treatment for HCC and the serum albumin level were the first and second distinguishing factors between the Alive and Deceased groups. A decision-tree algorithm revealed that the best profile comprised treatment with hepatectomy or radiofrequency ablation and a serum albumin level ≥3.7 g/dL (Group 1). The second-best profile comprised treatment with hepatectomy or radiofrequency ablation and serum albumin levels <3.7 g/dL (Group 2). The 5-year overall survival rate was significantly higher in the Group 1 than in the Group 2. Thus, we demonstrated that curative treatment for HCC and serum albumin level >3.7 g/dL was the best prognostic profile for NAFLD-HCC patients. This novel prognostic algorithm for patients with NAFLD-HCC could be used for clinical management.

## Introduction

Hepatocellular carcinoma (HCC) is the third-most common cause of cancer-related death worldwide^1^. Many etiologies of HCC have been identified, including hepatitis B virus (HBV) infection, hepatitis C virus (HCV) infection, and excess alcohol intake. Recently, however, non-alcoholic fatty liver disease (NAFLD), which affects increasing numbers of patients in both Western countries and Asia, has become an exceptionally common risk factor for HCC^2,3^. We previously demonstrated that patients with NAFLD-related HCC (NAFLD-HCC) and those with alcoholic liver disease-related HCC had similarly poor prognoses, although the prevalence of liver cirrhosis is significantly lower among the NAFLD-HCC group^4^.

The prognosis of patients with HCC is influenced by various tumor-, host-, and treatment-related factors. For example, the tumor stage at diagnosis, vascular invasion, HCC recurrence, and distant metastasis are well established prognostic factors^5–7^. In addition, hepatic function, as assessed based on the serum albumin and bilirubin levels, and the presence of concomitant complications of obesity and diabetes influence the outcomes of patients with HCC^8–10^. Finally, therapies such as hepatic resection, radiofrequency ablation, transarterial chemoembolization, and sorafenib affect the prognosis of patients with HCC^11–13^. Although interactions among these factors influence prognosis, their relative contributions remain unclear.

A data mining analysis is a computer learning approach in which artificial intelligence is used to reveal factors and interactions between variables from large data sets, even if no *a priori* hypothesis has been imposed^14^. The benefits of this approach include the discovery of hidden factors/profiles and the provision of additional information that cannot be identified through a logistic regression analysis, and the results could be used to make stepwise decisions about disease management^15^. A random forest analysis is a data mining technique used to identify factors that distinguish between case and control groups. This type of analysis is associated with a high level of predictive accuracy and can be used to estimate the relative importance of each factor^16^. Additionally, decision tree analysis data mining techniques identify priorities used to reveal a series of classification rules^17,18^. This type of analysis classifies data sets of groups using profiles that comprise multiple factors. Recently, these data mining techniques have been used to investigate prognostic factors for pancreatic cancer^19^, breast cancer^20^, and leukemia^21^. To our knowledge, however, these newer statistical techniques have never been used to investigate the prognosis of patients with NAFLD-HCC.

The of this study was to investigate the factors associated with the prognosis of NAFLD-HCC patients using a random forest analysis. We additionally investigated profiles associated with prognosis using a decision tree analysis.

## Results

### Baseline characteristics and comparisons of the Alive and Deceased groups

The baseline patient characteristics and comparisons of the Alive and Deceased groups are summarized in Table 1. Patents in the Alive group were significantly younger than those in the Deceased group. The HCC size and number and serum AFP and DCP levels were significantly lower in the Alive group than in the Deceased group (Table 1). Furthermore, a significantly higher number of NAFLD-HCC patients were treated with hepatic resection in the Alive group, than that in the Deceased group. The serum albumin levels were significantly higher in the Alive group than in the Deceased group (Table 1); however, no significant difference was seen in HbA1c values, platelet counts, and serum levels of total bilirubin and total cholesterol between the two groups (Table 1). HCC is the main cause of death and liver-related death occupied 84.7% of all causes of death (Table 1).

**Table 1:** Enrolled Patient characteristics and comparison of the Alive and Deceased groups.

	Reference Value	All subjects (n = 247)		Deceased group (n = 111)		Alive group (n = 136)		P
		Median (IQR)	Range (min–max)	Median (IQR)	Range (min–max)	Median (IQR)	Range (min–max)	
Age (years)	N/A	71.0 (65.0–78.0)	41.0–89.0	73.0 (67.0–78.0)	49.0–89.0	70.0 (64.0–77.0)	41.0–89.0	0.0264
Sex (female/male)	N/A	86/161 (34.8%/65.2%)	N/A	32/79 (28.8%/71.2%)	N/A	54/82 (39.7%/60.3%)	N/A	0.0731
Body mass index	18.5–24.9	26.0 (23.4–29.5)	15.3–40.6	25.3 (22.6–29.2)	15.3–37.1	26.6 (23.9–29.9)	15.3–40.6	0.1373
Smoking (pack-year)	N/A	0.0 (0.0–18.0)	0.0–200.0	0.0 (0.0–13.5)	0.0–120.0	0.0 (0.0–23.5)	0.0–200.0	0.5659
Diabetes mellitus (Yes/No)	N/A	165/82 (66.8%/33.2%)	N/A	75/36 (67.6%/32.4%)	N/A	90/46 (66.2%/33.8%)	N/A	0.8173
Hypertension (Yes/No)	N/A	151/96 (61.1%/38.9%)	N/A	69/42 (62.2%/37.8%)	N/A	82/54 (60.3%/39.7%)	N/A	0.7644
**HCC variables**								
Size of HCC (mm)	N/A	39.5 (21.0–60.0)	5.0–180.0	44.0 (25.0–70.0)	7.0–180.0	33.0 (20.0–55.0)	5.0–150.0	0.0078
Number of HCC	N/A	1.0 (1.0–3.0)	1.0–20.0	2.0 (1.0–5.0)	1.0–20.0	1.0 (1.0–2.0)	1.0–20.0	<0.0001
TNM stage (I/II/III/IV)	N/A	42/104/66/35 (17%/42.1%/26.7%/14.2%)	N/A	12/44/28/27 (10.8%/39.7%/25.2%/24.3%)	N/A	30/60/38/8 (22.0%/44.1%/28.0%/5.9%)	N/A	0.0001
AFP (ng/mL)	<8.7	9.0 (5.0–47.9)	0.5–496100.0	14.0 (5.0–156.9)	0.5–496100	8.0 (5.0–18.0)	1.5–466790	0.0217
DCP (mAU/mL)	<40	168.0 (27.0–1553.0)	8.0–1402600.0	324.0 (48.0–6060)	8.0–1402600	90.5 (22.0–193890)	10.0–193890	0.0006
Hepatic resection/RFA/TACE/Others/BSC	N/A	84/42/80/27/14 (34%/17%/32%/11%/6%)	N/A	25/14/42/20/10 (22%/13%/38%/18%/9%)	N/A	59/28/38/7/4 (43%/21%/28%/5%/3%)	N/A	<0.0001
**Biochemical examinations**								
Hemoglobin (g/dL)	13.7–16.8	13.0 (11.5–14.3)	6.0–16.9	12.3 (11.1–14.1)	8.1–16.8	13.3 (11.9–14.5)	6.0–16.9	0.0211
Platelet count (x 10^3^/mm^3^)	13.1–36.2	14.0 (9.5–19.8)	3.2–49.6	15.3 (9.7–20.9)	3.8–49.6	13.7 (9.3–19.7)	3.2–41.7	0.3125
AST (IU/L)	13–30	43.0 (31.8–59.2)	10.0–941.0	46.0 (31.0–75.0)	10.0–441.0	41.0 (31.5–56.5)	13.0–941.0	0.0825
ALT (IU/L)	10–30	36.0 (26.0–52.0)	7.0–1136.0	38.0 (26.0–53.0)	9.0–218.0	36.0 (25.0–51.0)	7.0–1136.0	0.8376
Lactate dehydrogenase (IU/L)	119–229	221.0 (184.0–265.0)	95.0–1926.0	236.0 (194.0–287.0)	137.0–1926.0	206.5 (175.8–246.5)	95.0–1926.0	0.0017
ALP (IU/L)	115–359	329.0 (256.0–450.0)	98.0–2 492.0	376.5 (270.0–508.0)	98.0–2492	303.0 (240.5–390.0)	129.0–1850.0	0.0007
GGT (IU/L)	13–64	81.0 (44.5–165.0)	11.0–118.6.0	109.5 (49.0–237.8)	11.0–944.0	70.0 (40.0–125.0)	18.0–1186	0.0011
Albumin (g/dL)	4.1–5.1	3.9 (3.4–4.2)	2.0–5.2	3.6 (3.3–4.1)	2.0–5.0	4.0 (3.6–4.3)	2.5–5.2	0.0002
PT (international normalized ratio)	0.85–1.15	1.1 (1.0–1.2)	0.8–1.9	1.1 (1.0–1.2)	0.9–1.89	1.1 (1.0–1.2)	0.8–1.6	0.7130
Total bilirubin (mg/dL)	0.40–1.20	0.9 (0.6–1.2)	0. 1–13.0	0.8 (0.6–1.2)	0.1–13.0	0.9 (0.6–1.2)	0.2–4.1	0.2758
Total cholesterol (mg/dL)	142–219	168.5 (148.0–196.0)	78.0–399.0	100.0 (75.8–131.5)	30.0–525.0	101.0 (79.0–136.0)	34.0–513.0	0.6247
High-density lipoprotein cholesterol (mg/dL)	40–96	46.0 (36.0–54.8)	16.0–94.0	44.0 (32.0–54.0)	16.0–84.0	46.0 (38.0–56.0)	17.0–94.0	0.1538
Low-density lipoprotein cholesterol (mg/dL)	70–139	106.0 (81.0–126.8)	44.0–431.0	111.0 (81.0–131.0)	44.0–284.0	98.0 (79.0–126.0)	47.0–431.0	0.2289
Triglyceride (mg/dL)	40–149	101.0 (77.0–133.0)	30.0–525.0	136 (126–163)	79–207	136 (121–162)	87–188	0.9131
Fasting blood sugar (mg/dL)	70–109	117.0 (102.0–143.0)	26.0–396.0	114.0 (97.0–148.0)	26.0–396.0	119.0 (104.0–142.0)	75.0–266.0	0.6336
HbA1c (%)	4.3–5.8	6.0 (5.0–7.0)	4.0–11.0	6.0 (5.0–7.0)	4.0–11.0	6.0 (5.0–7.0)	4.0–11.0	0.2957
BUN (mg/dL)	8.0–20.0	15.0 (12.0–19.0)	3.5–99.9	15.0 (12.0–19.0)	3.5–99.9	15.1 (12.0–19.0)	5.5–79.9	0.6730
Creatinine (mg/dL)	0.65–1.07	0.8 (0.6–0.9)	0.1–17.0	0.8 (0.6–0.9)	0.2–13.0	0.75 (0.6–0.9)	0.1–17.0	0.5739
Iron (μg/dL)	54–200	95.0 (63.0–129.2)	14.0–288.0	83.0 (50.5–123.5)	19.0–277.0	105.0 (76.0–142.0)	14.0–288.0	0.0071
Ferritin (ng/mL)	39.4–340	169.4 (60.2–360.7)	0.0–1528.0	159.5 (47.5–411.2)	0.0–1528.0	188.0 (81.8–349.6)	3.7–1170.0	0.7934
Anti-HBc antibody (Negative/Positive/No test)	Negative	127/58/72 (49%/23%/28%)	N/A	52/29/23 (53%/26%/21%)	N/A	75/29/32 (55%/23%/28%)	N/A	0.3659
Cause of death				HCC 79 (71.2%) Liver failure 14 (12.6%) Esophageal varices 1 (0.9%) Cardiovascular disease 4 (3.6%) Renal failure 2 (1.8%) Pancreatic cancer 1 (0.9%) Leukemia 1 (0.9%) Unknown 9 (8.1%)				

Note. Data are expressed as medians (interquartile ranges [IQR]), ranges, or numbers. “HCC treatment: Others” includes sorafenib, radiotherapy, and hepatic arterial infusion chemotherapy. Abbreviations: N/A, not applicable; HCC, hepatocellular carcinoma; TNM, tumor-node-metastasis; AFP, alpha-fetoprotein; DCP, des-γ-carboxy prothrombin; RFA, radiofrequency ablation; TACE, transarterial chemoembolization; BSC, best supportive care; AST, aspartate aminotransferase; ALT, alanine aminotransferase; ALP, alkaline phosphatase; GGT, gamma-glutamyl transpeptidase; PT, prothrombin activity; HbA1c, hemoglobin A1c; BUN, blood urea nitrogen; HBc, hepatitis B core.

### Overall analysis

A multivariate analysis showed that HCC treatment: others and BSC, age, and TNM stage III or IV were independent risk factors related to the prognosis of patients with NAFLD-HCC (Table 2). Meanwhile, the serum albumin level and body mass index (BMI) were found to be independent negative risk factors (Table 2).

**Table 2:** Cox regression model analysis of the prognosis of NAFLD-HCC patients.

	Coefficient	Hazard Ratio	95% Confidence Interval	P
HCC treatment: Others	0.78	2.18	1.11–4.28	0.02
HCC treatment: RFA	0.06	1.06	0.53–2.11	0.87
HCC treatment: TACE	0.49	1.64	0.98–2.75	0.06
HCC treatment: BSC	2.33	10.25	4.59–22.90	<0.0001
Albumin	−1.18	0.31	0.22–0.44	<0.0001
Age	0.05	1.05	1.02–1.07	0.00019
BMI	−0.06	0.94	0.90–0.99	0.01
TNM: stage II	0.48	1.62	0.84–3.11	0.15
TNM: stage III	0.75	2.11	1.06–4.23	0.03
TNM: stage IV	1.52	4.55	2.11–9.81	0.00011

Note. “HCC treatment: Others” includes sorafenib, radiotherapy, and hepatic arterial infusion chemotherapy. Abbreviations: NAFLD, non-alcoholic fatty liver disease; HCC, hepatocellular carcinoma; RFA, radiofrequency ablation; TACE, transarterial chemoembolization; BSC, best supportive care; TNM, tumor-node-metastasis.

**Figure 1:**
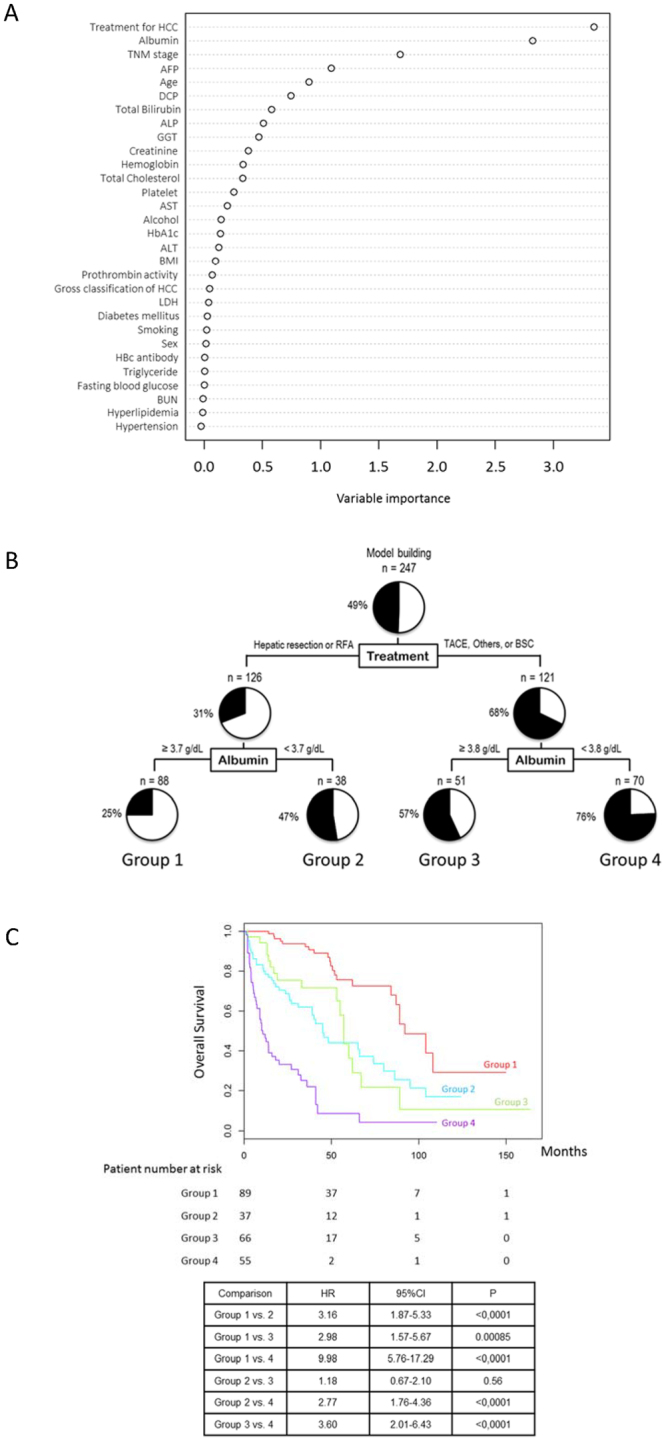
Factors/profiles associated with prognosis in the overall cohort of patients with NAFLD-HCC. (**A**) Random forest analysis. Data are expressed as variable importance. (**B**) Decision-tree algorithm. Patients with NAFLD-HCC are classified according to the indicated cut-off value of each factor. The pie graphs indicate the proportions of alive (white) and deceased patients (black). (**C**) Kaplan–Meier analysis. Abbreviations: NAFLD, non-alcoholic fatty liver disease; TNM, tumor-node-metastasis; AFP, alpha-fetoprotein; DCP, des-γ-carboxy prothrombin; ALP, alkaline phosphatase; GGT, gamma-glutamyl transpeptidase; AST, aspartate aminotransferase; HbA1c, hemoglobin A1c; ALT, alanine aminotransferase; BMI, body mass index; HCC, hepatocellular carcinoma; LDH, lactate dehydrogenase; HBc, hepatitis B core; BUN, blood urea nitrogen.

A random forest analysis demonstrated that treatment for HCC, serum albumin level, and TNM stage were the first, second, and third distinguishing factors, respectively, between the Alive and Deceased groups (Fig. 1A).

A decision-tree algorithm with 2 divergence variables was created to classify 4 profiles of patients (Fig. 1B). Treatment for HCC was the first variable in the initial classification. Among patients treated with hepatic resection or RFA, a serum albumin level ≥3.7 g/dL was the second-division variable in this classification. The serum albumin level was also the second-division variable among patients treated with TACE, other modalities, or BSC. As shown in Fig. 1B, the mortality rate of patients treated with hepatic resection or RFA and presenting with a serum albumin level ≥3.7 g/dL (Group 1) was 25.0% (22/88). By contrast, the mortality rate of patients treated with TACE, other modalities, or BSC and presenting with serum albumin levels <3.8 g/dL (Group 4) was 75.7% (53/70).

A Kaplan–Meier analysis yielded respective 1-, 3-, and 5-year survival rates of 100%, 92.3%, and 75.6% in Group 1 and 46.5%, 22.0%, and 9.0% in Group 4. Significant differences in overall survival were observed between Groups 1 and 4 (HR = 9.98, 95% CI: 5.76–17.29, P < 0.0001) (Fig. 1C).

### Stratification analysis according to TNM stage of HCC

A stratification analysis was performed according to the TNM stage of HCC. In each stage, the prognostic factors and profiles were analyzed using exploratory analyses including random forest analysis and decision tree analysis. NAFLD-HCC patients were classified into the group according to the results of the decision tree analysis and differences in survival rate among groups were analyzed by Kaplan–Meier analysis.

### TNM stage I

**Figure 2:**
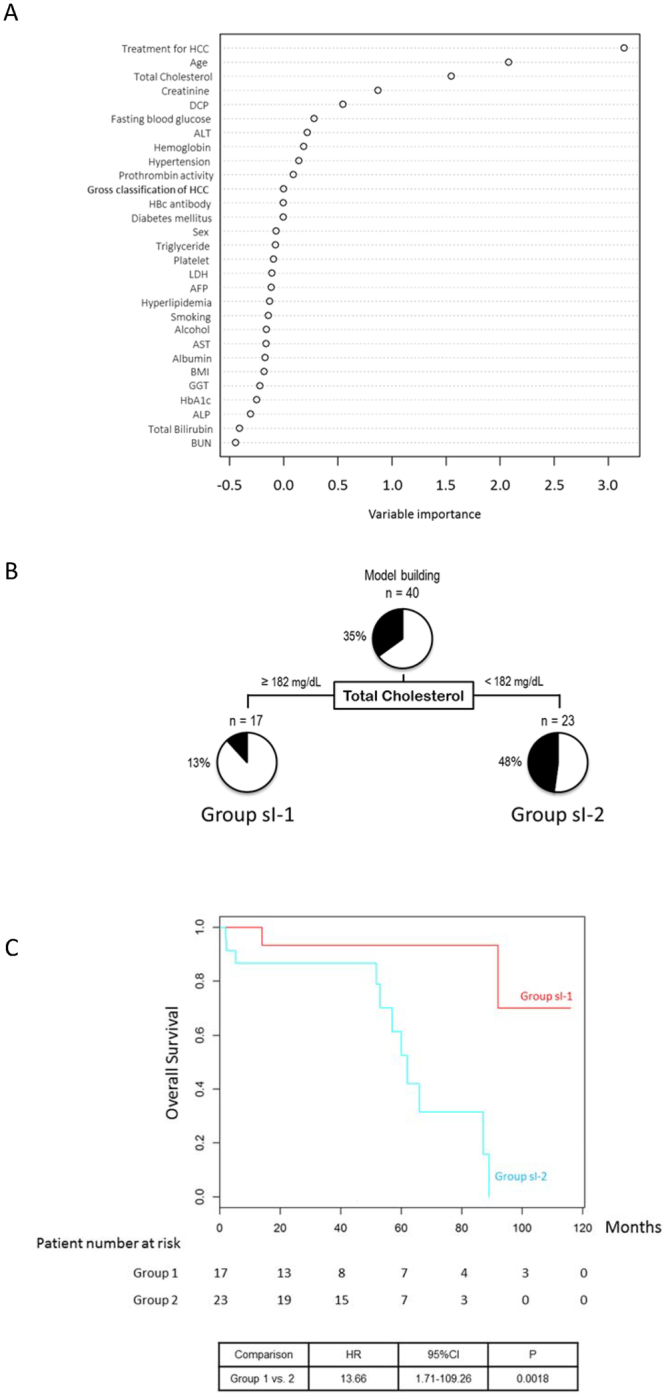
Factors/profiles associated with the prognosis of TNM stage I HCC patients with NAFLD. (**A**) Random forest analysis. Data are expressed as variable importance. (**B**) Decision-tree algorithm. Patients with NAFLD-HCC are classified according to the indicated cut-off value for each factor. The pie graphs indicate the proportions of alive (white) and deceased patients (black). (**C**) Kaplan–Meier analysis. Abbreviations: NAFLD, non-alcoholic fatty liver disease; DCP, des-γ-carboxy prothrombin; ALT, alanine aminotransferase; HCC, hepatocellular carcinoma; HBc, hepatitis B core; LDH, lactate dehydrogenase; AFP, alpha-fetoprotein; AST, aspartate aminotransferase; BMI, body mass index; GGT, gamma-glutamyl transpeptidase; HbA1c, hemoglobin A1c; ALP, alkaline phosphatase; BUN, blood urea nitrogen.

A multivariate analysis identified the prothrombin activity and serum AST levels as independent prognostic factors for patients with TNM stage 1 NAFLD-HCC (Table 3). Here, a random forest analysis demonstrated that the treatment of HCC, age, and serum total cholesterol level were the first, second, and third distinguishing factors between the Alive and Deceased groups (Fig. 2A). Next, a decision-tree algorithm was created using only the total cholesterol level (Fig. 2B). Among patients with a total cholesterol level ≥182 mg/dL (Group sI-1), the mortality rate was 13% (2/17). By contrast, the mortality rate among patients with a total cholesterol level <182 mg/dL (Group sI-2) was 48% (11/23). A Kaplan–Meier analysis yielded respective 1-, 3-, and 5-year survival rates of 100.0%, 93.3%, and 93.3% in Group sI-1 and 86.7%, 86.7%, and 52.6% in Group sI-2. Significant differences in survival were observed between Groups 1 and 2 (HR = 13.66, 95% CI: 1.71–109.26, P = 0.0018) (Fig. 2C).

**Table 3:** Stratification analysis of the prognosis of NAFLD-HCC patients according to TNM stage.

	Coefficient	Hazard Ratio	95% Confidence Interval	P
Stage I				
Age	2.35	10.44	9.80^e−01^–1.10^e+02^	0.05
Prothrombin activity	59.81	9.45 ^e+25^	1.43^e+01^–6.24^e+50^	0.04
Platelet count	−2.03	0.13	1.00^e−02^–1.21^e+00^	0.07
HbA1c	6.83	924.21	5.20^e−01^–1.66^e+06^	0.07
AST	−0.32	0.73	5.40^e−01^–9.80^e−01^	0.04
No complication of diabetes mellitus	11.49	97972.1	3.10^e−01^–3.10^e+10^	0.08
LDH	−0.02	0.98	9.50^e−01^–1.00^e+00^	0.09
Stage II				
Albumin	−1.31	0.27	0.16–0.46	<0.0001
Age	0.07	1.07	1.03–1.12	0.0008
Stage III				
Albumin	−2.20	0.11	0.05–0.27	<0.0001
BMI	−0.10	0.90	0.83–0.99	0.03
Stage IV				
Albumin	−1.75	0.17	0.06–0.46	0.0005
DCP	0.00003	1.00	1.00–1.00	<0.0001
Creatinine	0.87	2.38	1.14–8.53	0.0017
Positive result of HBc antibody	1.14	3.12	0.76–0.97	0.03
BMI	−0.15	0.86	0.05–0.27	0.01
HCC treatment: Others	1.99	7.32	1.04–51.45	0.05
HCC treatment: TACE	1.61	5.01	0.70–35.75	0.03
HCC treatment: BSC	3.80	44.55	4.80–413.88	0.00084
LDH	0.004	1.00	1.00–1.01	0.02

Note: “HCC treatment: Others” includes sorafenib, radiotherapy, and hepatic arterial infusion chemotherapy. Abbreviations: NAFLD, non-alcoholic fatty liver disease; HbA1c, hemoglobin A1c; AST, aspartate aminotransferase, LDH; lactate dehydrogenase, BMI, body mass index; DCP, des-γ-carboxy prothrombin; HBc, hepatitis B core; TACE, transarterial chemoembolization; BSC, best supportive care; HCC, hepatocellular carcinoma.

### TNM stage II

**Figure 3:**
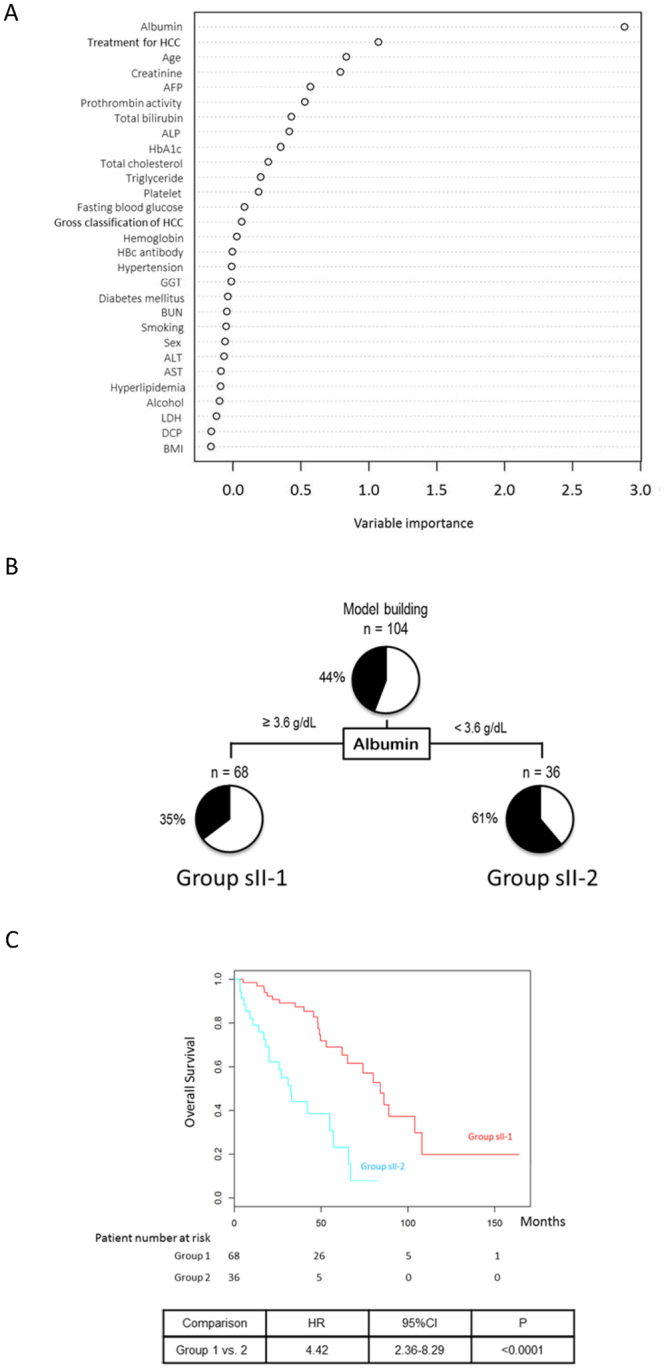
Factors/profiles associated with the prognosis of TNM stage II HCC patients with NAFLD. (**A**) Random forest analysis. Data are expressed as variable importance. (**B**) Decision-tree algorithm. Patients with NAFLD-HCC are classified according to the indicated cut-off value for each factor. The pie graphs indicate the proportions of alive (white) and deceased patients (black). (**C**) Kaplan–Meier analysis. Abbreviations: NAFLD, non-alcoholic fatty liver disease; AFP, alpha-fetoprotein; ALP, alkaline phosphatase; HbA1c, hemoglobin A1c; HCC, hepatocellular carcinoma; HBc, hepatitis B core; GGT, gamma-glutamyl transpeptidase; BUN, blood urea nitrogen; ALT, alanine aminotransferase; AST, aspartate aminotransferase; LDH, lactate dehydrogenase; DCP, des-γ-carboxy prothrombin; BMI, body mass index.

A multivariate analysis identified the serum albumin level as an independent negative risk factor and age as an independent risk factor among patients with TNM stage II NAFLD-HCC (Table 3). Here, the serum albumin level remained a first distinguishing factor between the Alive and Deceased groups in a random forest analysis (Fig. 3A). A decision-tree algorithm based only on the serum albumin level was created and used to classify 2 groups of patients (Fig. 3B). Accordingly, the mortality rate among patients with a serum albumin level ≥3.6 g/dL (Group sII-1) was 35% (24/68). By contrast, the mortality rate among those with a serum albumin level <3.6 g/dL (Group sII-2) was 61% (22/36). A Kaplan–Meier analysis yielded respective 1-, 3-, and 5-year survival rates of 98.5%, 87.4%, and 69.0% in Group sII-1 and 79.0%, 44.1%, and 23.1% in Group sII-2, respectively. These differences in survival between Group 1 and 2 were significant (HR = 4.42, 95% CI: 2.36–8.29, P < 0.0001) (Fig. 3C).

We also performed a propensity score matching analysis to reduce selection bias and confounding factors by calculating the propensity score consisted of age, sex, BMI, HCC treatment, platelet count, total bilirubin level, and presence of diabetes mellitus and hypertension (Supplementary Table 1). After the propensity score matching, a Kaplan–Meier analysis yielded respective 1-, 3-, and 5-year survival rates of 87.5%, 50.0%, and 37.5% in Group sII-1 and 66.7%, 13.3%, and 0.0% in Group sII-2, respectively. The difference in survival between Group sII-1 and Group sII-2 was significant (HR = 6.00, 95% CI: 4.50–8.11, P < 0.0001) (Supplementary Figure 1).

### TNM stage III

**Figure 4:**
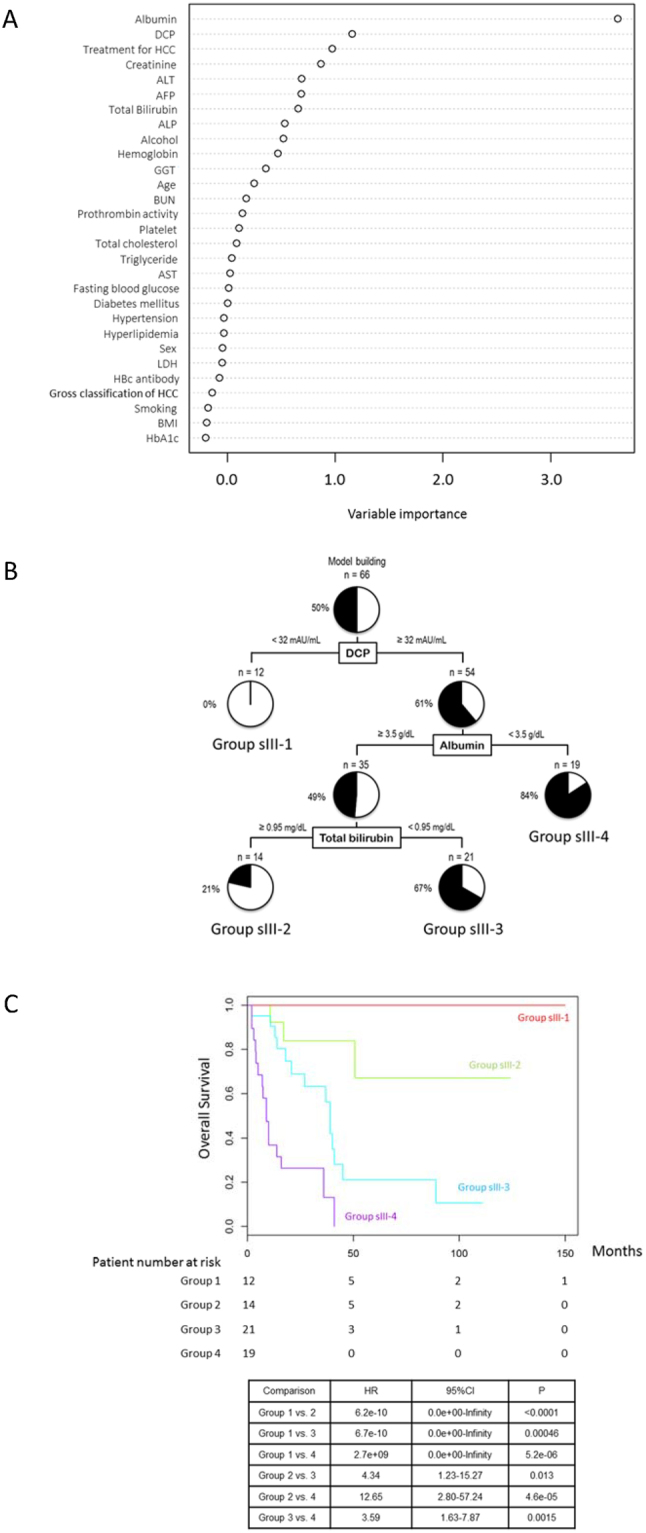
Factors/profiles associated with the prognosis of TNM stage III HCC patients with NAFLD. (**A**) Random forest analysis. Data are expressed as variable importance. (**B**) Decision-tree algorithm. Patients with NAFLD-HCC are classified according to the indicated cut-off value for each factor. The pie graphs indicate the proportions of alive (white) and deceased patients (black). (**C**) Kaplan–Meier analysis. Abbreviations: NAFLD, non-alcoholic fatty liver disease; DCP, des-γ-carboxy prothrombin; ALT, alanine aminotransferase; AFP, alpha-fetoprotein; ALP, alkaline phosphatase; GGT, gamma-glutamyl transpeptidase; BUN, blood urea nitrogen; AST, aspartate aminotransferase; LDH, lactate dehydrogenase; HBc, hepatitis B core; HCC, hepatocellular carcinoma; BMI, body mass index; HbA1c, hemoglobin A1c.

A multivariate analysis identified the serum albumin level and BMI as independent negative risk factors among patients with TNM stage III NAFLD-HCC (Table 3). A random forest analysis identified the serum albumin level as the first distinguishing factor between the Alive and Deceased groups (Fig. 4A). A decision-tree algorithm was created with 3 divergence variables and used to classify 4 patient profiles (Fig. 4B). Here, DCP was used as the first variable in the initial classification. Among patients with a DCP level >32 mAU/L, the second variable was the serum albumin level. Among patients with a serum albumin level >3.5 g/dL, the third variable was the serum bilirubin level. Here, all patients with a DCP level <32 mAU/mL (Group sIII-1, 12/12) remained alive. By contrast, the mortality rate among patients with a DCP level >32 mAU/mL and a serum albumin <3.5 g/dL (Group sIII-4) was 78.9% (15/19). According to Kaplan–Meier analysis, the respective 1- and 3-year survival rates were 100% and 100% in Group sIII-1 and 36.8% and 13.1% in Group sIII-4. Significant differences in survival were observed between Groups 1 and 4 (HR = 2.7e^+09^, 95% CI: 0.0e^+00^–Infinity, P = 5.2e^−06^) (Fig. 4C).

### TNM stage IV

**Figure 5:**
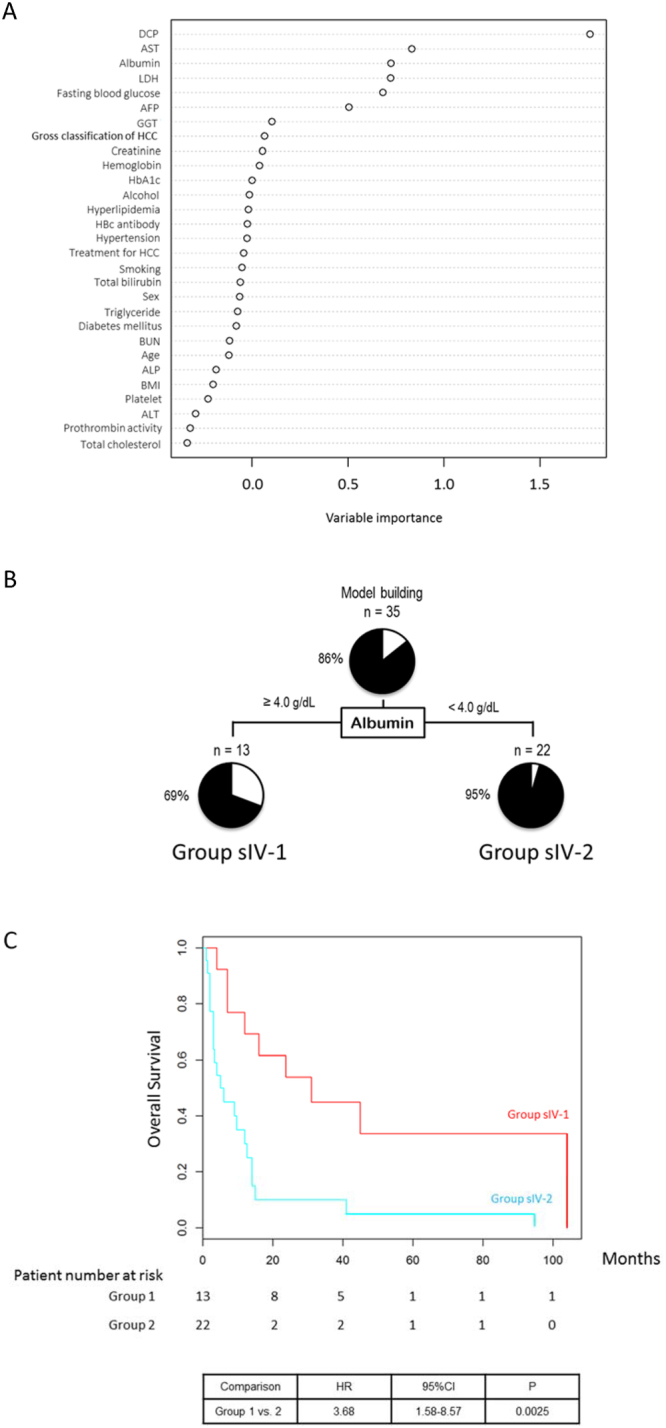
Factors/profiles associated with the prognosis of TNM stage IV HCC patients with NAFLD. (**A**) Random forest analysis. Data are expressed as variable importance. (**B**) Decision-tree algorithm. Patients with NAFLD-HCC are classified according to the indicated cut-off value for each factor. The pie graphs indicate the proportions of alive (white) and deceased patients (black). (**C**) Kaplan–Meier analysis. Abbreviations: NAFLD, non-alcoholic fatty liver disease; DCP, des-γ-carboxy prothrombin; AST, aspartate aminotransferase; LDH, lactate dehydrogenase; AFP, alpha-fetoprotein; GGT, gamma-glutamyl transpeptidase; HCC, hepatocellular carcinoma; HbA1c, hemoglobin A1c; HBc, hepatitis B core; BUN, blood urea nitrogen; ALP, alkaline phosphatase; BMI, body mass index; ALT, alanine aminotransferase.

A multivariate analysis identified the serum levels of DCP, creatinine, and LDH and positivity for the HBc antibody as independent prognostic factors among patients with TNM stage IV NAFLD-HCC (Table 3). The serum albumin level and BMI were identified as independent negative risk factors (Table 3). A random forest analysis identified the serum DCP, AST, and albumin levels as the first, second, and third distinguishing factors between the Alive and Deceased groups (Fig. 5A). A decision-tree algorithm was created based only on the serum albumin level and was used to classify 2 groups of patients (Fig. 5B). Although the mortality rate of patients with serum albumin levels of ≥4 g/dL (Group sIV-1) was 69% (9/13), this rate increased to 95% (21/22) among those with serum albumin levels <4 g/dL (Group sIV-2). A Kaplan–Meier analysis yielded respective 1-, 3- and 5-year survival rates of 69.2%, 44.9%, and 33.7% in Group sIV-1 and 30.0%,10.0%, and 5% in Group sIV-2. Significant differences in survival were observed between these groups (HR = 3.68, 95% CI: 1.58–8.57, P = 0.0025) (Fig. 5C).

## Discussion

We first applied an artificial intelligence-based approach to one of the largest NAFLD-HCC data sets to investigate the prognostic factors/profiles relevant to patients. Our study used a random forest analysis to demonstrate that treatment for HCC, the serum albumin level, and the TNM stage were significant prognostic factors among patients with NAFLD-HCC. A decision tree analysis revealed that a patient profile comprising curative treatment for HCC and a serum albumin level >3.7 g/dL was associated with a better prognosis. Moreover, both random forest analyses and data mining analyses stratified by TNM stage revealed that the serum albumin level was a prognostic factor for patients with stage II–IV NAFLD-HCC.

Although the benefits of data mining analysis include the discovery of hidden factors/profiles with high predictive accuracy, one obstacle to this type of approach is the requirement for a large data set; therefore, we used the large data sets from JSG-NAFLD (n = 247). The clinical features of NAFLD-HCC in this study were similar to those in a previous report of another large data set study from the HCC-NAFLD Italian Study Group (n = 145)^22^. In addition, more than 95% of enrolled patients in our study had data for all variables, including AFP and DCP, thus confirming the reliability of our data sets. Moreover, none of the NAFLD-HCC patients enrolled in this study had undergone liver transplantation for reasons including advanced HCC, lack of a donor, age, or religious objections, which allowed us to discern the natural history of NAFLD-HCC.

Most HCCs arise in the context of chronic liver diseases with various etiologies, including chronic HBV/HCV infection, alcohol consumption, and NAFLD. For patients with HBV-related HCC, nucleotide analog therapy is known to improve prognosis after curative cancer treatment^23^. Similarly, for patients with HCV-related HCC, interferon-based treatment may improve prognosis by ameliorating the liver reserve of infection after curative treatment for HCC^24^. Therefore, treatment for the underlying liver disease or dysfunction, in addition to curative treatment of the primary tumor, can improve patient outcomes. However, little is known about the prognostic profiles of patients with NAFLD-HCC. In this study, we first applied data mining techniques and identified better prognoses with a profile comprising curative treatment for HCC and a serum albumin level >3.7 g/dL. Although obesity and type 2 diabetes mellitus have been identified as potent risk factors for HCC in patients with NAFLD^25,26^, our algorithm is specific for NAFLD patients, which suggest that the liver reserve is a more important prognostic risk factor than obesity or type 2 diabetes mellitus.

The tumor stage is widely considered an absolute categorical factor for survival in patients with primary liver tumors. Although various tumor staging systems have been used, the TNM system is reported to predict the prognoses of patients with both advanced and early tumors^27^. Therefore, we performed both random forest and decision tree analyses stratified by TNM stage and again found that the serum albumin level influenced prognosis, particularly among those with TNM stage II–IV disease. Recently, the albumin-bilirubin grade, an index of the functional liver reserve, was shown to predict prognosis across all stages of HCC in a study wherein 93% of patients had virus-related cancers^28^. The present results are consistent with those of the earlier study, and the liver functional reserve seems to be a universal prognostic factor for most HCC patients, regardless of the chronic liver disease etiology.

In our study, serum albumin level was a prognostic factor for patients with NAFLD-HCC, indicating that hepatic fibrosis is the prognostic factor. In addition, our findings suggested that serum albumin level had higher impact on the prognosis than other hepatic parameters including platelet count, prothrombin activity, total cholesterol, and bilirubin in both the random forest and decision-tree analyses. We also performed a propensity score matching. Even after the propensity score matching, the survival rate of patients with a serum albumin level ≥3.6 g/dL was significantly higher than patients with a serum albumin level <3.6 g/dL. These findings also suggest that serum albumin has unique implication other than a hepatic fibrosis-related factor. The decreased albumin may be caused by low intake of protein and/or an oxidative stress-induced degradation of albumin^29^. Serum albumin exerts anti-oxidative activity by harboring a disulfide-bonded cysteine at the thiol of Cys34 and the oxidized albumin is degraded by endogenous proteases^29^. Albumin is also known to bind with cisplatin at the III domain to enhance the anti-tumor activity of this drug^12^. In fact, the baseline serum albumin level is a prognostic factor in patients with various malignancies, including those of the colon, lung, and breast cancer^30–32^. Moreover, Nojiri *et al*. reported that albumin suppresses the proliferation of HCC cell lines by upregulating the expression of p21 and p57 and consequently increasing the G0/G1 cell population^33^. Thus, serum albumin level may reflect degree of oxidative stress and anti-tumor activity in patients with NAFLD.

A limitation of this study is the reliability of this algorithm. Since we did not validate the algorithm, further prospective study is required to test the reliability of this algorithm. We also must be cautious in the interpretation for the results the Cox regression model analysis. In this study, we proposed a novel prognostic algorithm based on treatment for HCC and the serum albumin level. In addtion, age, BMI, and TNM stage were identified as independent prognostic factors in the Cox regression model analysis. Thus, these independent factors should also be paid attention for the management of patients with NAFLD-HCC.

In conclusion, this nationwide data mining analysis-based study identified treatment for HCC, the serum albumin level, and the TNM stage as significant long-term prognostic factors among patients with NAFLD-HCC. We identified a profile comprising curative treatment for HCC and a serum albumin level >3.7 g/dL as predictive of a better prognosis. Furthermore, we identified the serum albumin level as a prognostic factor for patients with stage II–IV HCC. These findings suggest that this novel prognostic algorithm could be used for the clinical management of patients with NAFLD-HCC.

## Subjects and Methods

### Study design and ethics

This retrospective study was designed in 2015 by the steering committee of the Japan Study Group of NAFLD (JSG-NAFLD) as a multicenter investigation of the prognosis of patients with NAFLD-HCC. This protocol conformed to the ethical guidelines of the 1975 Declaration of Helsinki, as reflected by the prior approval of the institutional review board of Kurume University School of Medicine, Tokyo Women’s Medical University, JA Hiroshima General Hospital, Hiroshima University, Sapporo Kosei General Hospital, Kochi Medical School, Kawasaki Medical School, Asahikawa Medical University, Nayoro City General Hospital, Yokohama City University School of Medicine, Oita University, Saga University, Nara City Hospital, Kyoto Prefectural University of Medicine, Aichi Medical University, National Center for Global Health and Medicine, Osaka University, Osaka City University, and Osaka City Juso Hospital. All experiments were performed in accordance with relevant guidelines and regulations. An opt-out approach was used to obtain informed consent from the patients, and personal information was protected during data collection.

### Subjects

A total of 247 consecutive patients diagnosed with NAFLD-HCC between 2000 and 2014 were registered from 17 medical institutions in Japan. Of these, 136 patients remained alive (Alive group) and 111 patients had died (Deceased group) at the censor time of this study (December 2014).

### Diagnosis of NAFLD and HCC

NAFLD-HCC was diagnosed according to the Clinical Practice Guidelines for NAFLD/nonalcoholic steatohepatitis (NASH) as follows^34^: (1) hepatic steatosis evaluated by liver biopsy, ultrasonography, computed tomography, or magnetic resonance imaging; (2) ethanol intake <20 g/day in women or <30 g/day in men; and (3) exclusion of other liver diseases, including HBV, HCV, autoimmune hepatitis, drug-induced liver disease, primary biliary cholangitis, primary sclerosing cholangitis, biliary obstruction, Wilson’s disease, and hemochromatosis.

HCC was diagnosed via histological examination or a combination of serum tumor makers such as α-fetoprotein (AFP) and des-γ-carboxy prothrombin (DCP), as well as imaging modalities such as ultrasonography, computed tomography, magnetic resonance imaging, and/or angiography according to the Japanese Clinical Practice guidelines for HCC: The Japan Society of Hepatology^35^.

### Inclusion and exclusion criteria

The following patient inclusion criteria were used: (1) NAFLD-HCC, (2) age >18 years, (3) no previous treatment for HCC, and (4) complete follow-up from the initial treatment for HCC until death or the study censor time (December 2014). The exclusion criteria were as follows: (1) a history of a malignant tumor other than HCC within the 5 years preceding the study and (2) participation in any drug trial.

### Data collection

Variables related to host, tumor, and treatment factors were retrospectively reviewed using clinical records. The following data were collected at the time of diagnosis of HCC: host factors, including age, sex, body mass index (BMI), smoking (pack-year), hemoglobin level, platelet count, fasting blood glucose level, hemoglobin A1c (HbA1c) level, prothrombin activity, and serum levels of aspartate aminotransferase (AST), alanine aminotransferase (ALT), lactate dehydrogenase (LDH), gamma-glutamyl transpeptidase (γ-GTP), alkaline phosphatase (ALP), albumin, total bilirubin, total cholesterol, high density lipoprotein-cholesterol, low density lipoprotein-cholesterol, triglyceride, blood urea nitrogen (BUN), creatinine, and hepatitis B core (HBc) antibody; tumor factors, including the size and number of HCC, serum levels of AFP and DCP, gross classification of HCC, and clinical staging (tumor-node-metastasis [TNM] classification) based on the criteria of the Liver Cancer Study Group of Japan^36^ (stage I, n = 40; stage II, n = 104; stage III, n = 66; stage IV, n = 35; lack of sufficient data for staging; n = 2); and treatment factors such as the selected treatment modality [hepatic resection, radiofrequency ablation (RFA), transarterial chemoembolization (TACE), others (sorafenib, radiotherapy, and hepatic arterial infusion chemotherapy), best supportive care (BSC)]. Treatments were selected according to the HCC guidelines of the Japan Society of Hepatology^37^.

The datasets generated during and/or analysed during the current study are available from the corresponding author on reasonable request.

### Definition of event and follow-up

In this study, an event was defined as death from any cause. After the initial treatment for HCC, patients were followed up until death or the study censor date through routine physical examinations, biochemical tests (including serum AFP and DCP levels), and abdominal imaging (including ultrasonography, computed tomography, or magnetic resonance imaging) according to the HCC guidelines of the Japan Society of Hepatology^37^. HCC patients treated with BSC were also followed up.

### Statistics

Data are expressed as numbers or means ± standard deviations. Differences between the two groups were analyzed using the Mann–Whitney *U* test. Factors or profiles associated with the prognosis of NAFLD-HCC patients were analyzed using data mining techniques. All statistical analyses were conducted by a biostatistician (AK). The statistical methods are described in detail below.

### Multivariate stepwise analysis

A Cox regression model was used to identify independent variables associated with the prognosis of NAFLD-HCC in a multivariate analysis. Based on our purpose, we didn’t conduct the univariate analysis. Explanatory variables were selected from variables listed in Table 1 by the stepwise manner minimizing the Bayesian information criterion as previously described^15^. Data were expressed as hazard ratios (HR) and 95% confidence intervals (CI).

### Random forest analysis

A random forest analysis was used to identify factors that distinguished between the Alive and Deceased groups on an ordinal scale, as previously described^15^. The variable importance (VI) value, which reflects the relative contribution of each variable to the model, was estimated by randomly permuting its values and recalculating the predictive accuracy of the model.

### Decision tree algorithm

A decision-tree algorithm was constructed to reveal profiles associated with the prognosis of NAFLD-HCC according to the instructions provided with the R software package (http://www.R-project.org/)^38^.

### Kaplan–Meier analysis

NAFLD-HCC patients were classified into the correspond group of the decision-tree algorithm. The overall survival of each group was estimated using the Kaplan–Meier method, and differences in survival between the groups were analyzed using the log-rank test.

All P values were 2-tailed, and a value <0.05 was considered statistically significant. The multivariate stepwise analysis, random forest analysis, decision tree analysis, and Kaplan–Meier analysis were performed using the R software package^38^.

## Electronic supplementary material

Supplementary Table 1
