# Supplementary material for: A Data Mining-based Prognostic Algorithm for NAFLD-related Hepatoma Patients: A Nationwide Study by the Japan Study Group of NAFLD
Source: Sci Rep. 2018 Jul 11;8:10434. doi: 10.1038/s41598-018-28650-0 (PMC6041283; doi:10.1038/s41598-018-28650-0)
Supplement: Supplementary file 1 — Supplementary Table 1 [file 41598_2018_28650_MOESM1_ESM.pdf]

Supplementary Information

A Data Mining-based Prognostic Algorithm for NAFLD-related Hepatoma Patients: A Nationwide Study by the Japan Study Group of NAFLD

Takumi Kawaguchi[1], Katsutoshi Tokushige[2], Hideyuki Hyogo[3], Hiroshi Aikata[4], Tomoaki Nakajima[5], Masafumi Ono[6], Miwa Kawanaka[7], Koji Sawada[8], Kento Imajo[10], Koichi Honda[11], Hirokazu Takahashi[12], Kohjiroh Mori[14], Saiyu Tanaka[14], Yuya Seko[15], Yuichi Nozaki[17], Yoshihiro Kamada[18], Hideki Fujii[19, 20], Atsushi Kawaguchi[21], Tetsuo Takehara[18], Mikio Yanase[17], Yoshio Sumida[16], Yuichiro Eguchi[13], Masataka Seike[11], Masato Yoneda[10], Yasuaki Suzuki[9], Toshiji Saibara[6], Yoshiyasu Karino[5], Kazuaki Chayama[4], Etsuko Hashimoto[2], Jacob George[22], Takuji Torimura[1]

1) Department of Medicine, Kurume University School of Medicine, Kurume, Japan.
2) Department of Internal Medicine and Gastroenterology, Tokyo Women's Medical University, Tokyo, Japan.
3) Department of Gastroenterology and Hepatology, JA Hiroshima General Hospital, Hatsukaichi, Japan.
4) Department of Gastroenterology and Metabolism, Applied Life Science, Institute of Biomedical and Health Sciences, Hiroshima University, Hiroshima, Japan.
5) Department of Hepatology, Sapporo Kosei General Hospital, Sapporo, Japan.
6) Department of Gastroenterology and Hepatology, Kochi Medical School, Nankoku, Japan.
7) Department of General Internal Medicine2, General Medical Center, Kawasaki Medical School, Okayama, Japan.
8) Division of Gastroenterology and Hematology/Oncology, Department of Medicine, Asahikawa Medical University, Asahikawa, Japan.

9) Department of Gastroenterology, Nayoro City General Hospital, Nayoro, Japan.
10) Department of Gastroenterology and Hepatology, Yokohama City University School of Medicine, Yokohama, Japan.
11) Department of Gastroenterology, Faculty of Medicine, Oita University, Yufu, Japan.
12) Internal Medicine, Saga University faculty of Medicine, Saga, Japan.
13) Liver Center, Saga University Hospital, Saga, Japan.
14) Center for Digestive and Liver Diseases, Nara City Hospital, Nara, Japan.
15) Department of Gastroenterology and Hepatology, Kyoto Prefectural University of Medicine, Kyoto, Japan.
16) Division of Hepatology and Pancreatology, Department of Internal Medicine, Aichi Medical University, Nagakute, Japan.
17) Department of Gastroenterology, National Center for Global Health and Medicine, Tokyo, Japan.
18) Department of Gastroenterology and Hepatology, Osaka University, Graduate School of Medicine, Suita, Japan.
19) Department of Hepatology, Osaka City University, Graduate School of Medicine, Osaka, Japan.
20) Department of Gastroenterology and Hepatology, Osaka City Juso Hospital, Osaka, Japan.
21) Center for Comprehensive Community Medicine Faculty of Medicine, Saga University, Saga, Japan.
22) Storr Liver Centre, Westmead Institute for Medical Research, Westmead Hospital and University of Sydney, Sydney, NSW, Australia.

Supplementary Table 1. Comparison of patient characteristics between Alive and Deceased groups in TNM stage II after a propensity score matching

	Reference Value	Deceased group (n = 17) Median (IQR)	Deceased group (n = 17) Range (min–max)	Alive group (n = 17) Median (IQR)	Alive group (n = 17) Range (min–max)	P
Matched variables						
Age (years)	N/A	73.0 (64.4–79.5)	54.0–89.0	71.0 (62.4–79.0)	60.0–86.0	0.5806
Sex (female/male)	N/A	8/9 (47.1%/52.9%)	N/A	6/11 (35.3%/64.7%)	N/A	0.4858
Body mass index	18.5–24.9	26.1 (24.0–29.3)	21.1–34.9	26.8 (23.0–29.9)	17.3–33.2	0.8358
Hepatic resection/RFA/TACE/Others/BSC	N/A	8/3/6/0/0 (47%/18%/35 %/0%/0%)	N/A	7/3/6/0/1 (41%/18%/35%/ 0%/6%)	N/A	0.7851
Platelet count (x $10^3/mm^3$)	13.1–36.2	15.7 (8.6–21.3)	6.2–41.7	16.0 (9.3–20.8)	7.2–39.1	0.8768
Total bilirubin (mg/dL)	0.40–1.20	0.7 (0.6–1.3)	0.3–1.9	0.6 (0.5–0.9)	0.3–4.8	0.3585
Diabetes mellitus (Yes/No)	N/A	13/4 (76.5%/23.5%)	N/A	11/6 (64.7%/35.3%)	N/A	0.570
Hypertension (Yes/No)	N/A	14/3 (82.4%/17.3%)	N/A	13/4 (76.5%/23.5%)	N/A	0.6715

Biochemical examinations (Unmatched variables)						
Hemoglobin (g/dL)	13.7–16.8	12.8 (11.2–14.5)	10.8–16.6	11.8 (10.7–13.1)	8.1–14.0	0.0652
AST (IU/L)	13–30	40.0 (28.5–49.0)	21.0–75.0	43.0 (25.5–94.0)	15.0–941.0	0.4798
ALT (IU/L)	10–30	35.0 (27.5–55.5)	11.0–90.0	28.0 (19.0–48.5)	10.0–1136.0	0.4718
Lactate dehydrogenase (IU/L)	119–229	196.5 (174.8–290.3)	143.0–318.0	223.0 (200.0–295.0)	160.0–1278.0	0.1546
ALP (IU/L)	115–359	278.0 (224.0–376.0)	153.0–650.0	387.0 (260.5–455.5)	125.0–1352.0	0.1736
GGT (IU/L)	13–64	52.0 (40.0–96.5)	16.0–244.0	67.0 (40.5–234.0)	11.0–480.0	0.5581
PT (international normalized ratio)	0.85–1.15	1.06 (1.00–1.18)	0.91–1.38	1.14 (1.05–1.40)	0.97–1.59	0.0990
Total cholesterol (mg/dL)	142–219	172.0 (155.0–207.5)	125.0–235.0	147.0 (118.0–182.0)	91.0–251.0	0.0716
High-density lipoprotein cholesterol (mg/dL)	40–96	47.5 (41.0–58.5)	27.0–81.0	46.9 (32.3–54.9)	26.0–92.9	0.6032
Low-density lipoprotein cholesterol (mg/dL)	70–139	108.5 (85.5–149.3)	66.0–246.0	80.0 (72.0–92.0)	44.0–144.0	0.0863
Triglyceride (mg/dL)	40–149	136.5 (78.3–180.5)	79–207	136 (121–162)	87–188	0.0506
Fasting blood sugar (mg/dL)	70–109	111.0 (109.0–129.0)	64.0–23.0	92.5 (58.0–128.5)	30.0–151.0	0.7055

HbA1c (%)	4.3–5.8	6.6 (5.5–7.1)	5.4–8.9	5.8 (5.2–6.8)	4.3–9.6	0.0964
BUN (mg/dL)	8.0–20.0	17.1 (11.5–21.1)	8.9–30.4	18.3 (11.3–27.8)	3.5–99.9	0.7175
Creatinine (mg/dL)	0.65–1.07	0.88 (0.71–0.96)	0.55–1.25	0.83 (0.59–1.08)	0.48–2.12	0.9051
Anti-HBc antibody (Negative/Positive/No test)	Negative	8/7/2 (47%/41%/12 %)	N/A	10/5/2 (59%/29%/12%)	N/A	0.4552

Note. Data are expressed as medians (interquartile ranges [IQR]), ranges, or numbers. “HCC treatment: Others” includes sorafenib, radiotherapy, and hepatic arterial infusion chemotherapy. Abbreviations: N/A, not applicable; HCC, hepatocellular carcinoma; TNM, tumor-node-metastasis; AFP, alpha-fetoprotein; DCP, des-γ-carboxy prothrombin; RFA, radiofrequency ablation; TACE, transarterial chemoembolization; BSC, best supportive care; AST, aspartate aminotransferase; ALT, alanine aminotransferase; ALP, alkaline phosphatase; GGT, gamma-glutamyl transpeptidase; PT, prothrombin activity; HbA1c, hemoglobin A1c; BUN, blood urea nitrogen; HBc, hepatitis B core.

Supplementary Figure 1

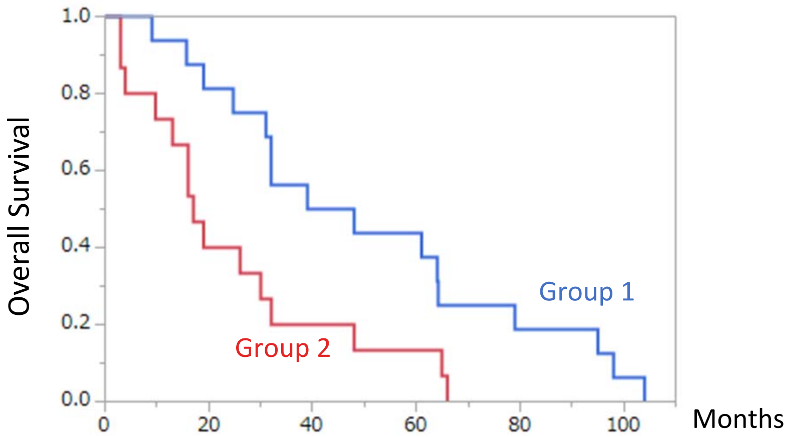

Patient number at risk

Group 1	17	14	9	7	3	1
Group 2	17	6	3	2	0	0

Comparison	HR	95%CI	P
Group 1 vs. 2	6.00	4.50-8.11	< 0.0001
